# Heptamagnesium bis­(phosphate) tetra­kis­(hydrogen phosphate) with strong hydrogen bonds: Mg_7_(PO_4_)_2_(HPO_4_)_4_

**DOI:** 10.1107/S1600536811036361

**Published:** 2011-09-14

**Authors:** Abderrazzak Assani, Mohamed Saadi, Mohammed Zriouil, Lahcen El Ammari

**Affiliations:** a Laboratoire de Chimie du Solide Appliquée, Faculté des Sciences, Université Mohammed V-Agdal, Avenue Ibn Battouta, BP. 1014, Rabat, Morocco

## Abstract

The title compound, Mg_7_(PO_4_)_2_(HPO_4_)_4_, was synthesized by the hydro­thermal method. The structure is based on a framework of edge- and corner-sharing MgO_6_ and MgO_4_(OH)_2_ octa­hedra, an MgO_5_ polyhedron, PO_4_ and PO_3_(OH) tetra­hedra. All atoms are in general positions except for one Mg atom, which is located on a crystallographic inversion centre. The OH groups, bridging Mg–(OH)–P, are involved in strong hydrogen bonds. Compounds with the general formula *M*_7_(PO_4_)_2_(HPO_4_)_4_ (*M* = Mg, Mn, Fe and Co) are all isostructural with their homologue arsenate Mg_7_(AsO_4_)_2_(HAsO_4_)_4_.

## Related literature

For background to metal phosphates, see: Viter & Nagornyi (2009 ▶); Clearfield (1988 ▶); Trad *et al.* (2010 ▶). For the hydro­thermal method, see: Assani *et al.* (2010 ▶, 2011**a* ▶,b* ▶). For isostructural compounds, see: Zhou *et al.* (2002 ▶); Riou *et al.* (1987 ▶); Rojo *et al.* (2002 ▶); Lightfoot & Cheetham (1988 ▶); Kolitsch & Bartu (2004 ▶).

## Experimental

### 

#### Crystal data

Mg_7_(PO_4_)_2_(HPO_4_)_4_
*M* *_r_* = 744.02
Triclinic, 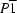

*a* = 6.4204 (5) Å
*b* = 7.8489 (4) Å
*c* = 9.4315 (5) Å
α = 104.442 (3)°
β = 108.505 (5)°
γ = 101.189 (8)°
*V* = 416.70 (4) Å^3^
*Z* = 1
Mo *K*α radiation
μ = 1.06 mm^−1^
*T* = 296 K
0.16 × 0.10 × 0.07 mm

#### Data collection

Bruker X8 APEX Diffractometer
Absorption correction: multi-scan (*SADABS*; Bruker, 2005 ▶) *T*_min_ = 0.881, *T*_max_ = 0.929
9421 measured reflections
1923 independent reflections
1715 reflections with *I* > 2σ(*I*)
*R*_int_ = 0.036

#### Refinement

*R*[*F* ^2^ > 2σ(*F* ^2^)] = 0.026
*wR*(*F* ^2^) = 0.072
*S* = 1.07
1923 reflections
169 parameters
H-atom parameters constrained
Δρ_max_ = 0.43 e Å^−3^
Δρ_min_ = −0.42 e Å^−3^

### 

Data collection: *APEX2* (Bruker, 2005 ▶); cell refinement: *SAINT* (Bruker, 2005 ▶); data reduction: *SAINT*; program(s) used to solve structure: *SHELXS97* (Sheldrick, 2008 ▶); program(s) used to refine structure: *SHELXL97* (Sheldrick, 2008 ▶); molecular graphics: *ORTEP-3 for Windows* (Farrugia, 1997 ▶) and *DIAMOND* (Brandenburg, 2006 ▶); software used to prepare material for publication: *WinGX* publication routines (Farrugia, 1999 ▶).

## Supplementary Material

Crystal structure: contains datablock(s) I. DOI: 10.1107/S1600536811036361/bt5638sup1.cif

Structure factors: contains datablock(s) I. DOI: 10.1107/S1600536811036361/bt5638Isup2.hkl

Supplementary material file. DOI: 10.1107/S1600536811036361/bt5638Isup3.cml

Additional supplementary materials:  crystallographic information; 3D view; checkCIF report

## Figures and Tables

**Table 1:** Hydrogen-bond geometry (Å, °)

*D*—H⋯*A*	*D*—H	H⋯*A*	*D*⋯*A*	*D*—H⋯*A*
O4—H4⋯O7^i^	0.86	1.61	2.460 (2)	172
O12—H12⋯O10^ii^	0.86	1.80	2.656 (2)	171

Symmetry codes: (i) 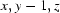; (ii) 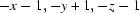.

**Fig. 1.**
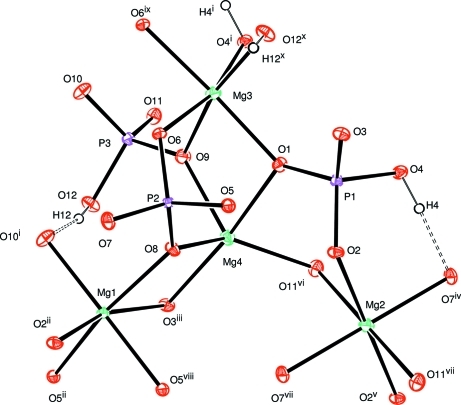
Partial plot of Mg7(PO4)2(HPO4)4 crystal structure. Displacement ellipsoids are drawn at the 50% probability level. Symmetry codes: (i) -x - 1, -y + 1, -z - 1; (ii) -x, -y + 1, -z; (iii) x - 1, y, z; (iv) x, y - 1, z; (v) -x, -y, -z; (vi) -x - 1, -y, -z - 1; (vii) x + 1, y, z + 1; (viii) -x, -y, -z - 1; (ix) -x, -y + 1, -z - 1; (x) x + 1, y, z; (xi) x, y + 1, z; (xii) x - 1, y, z - 1.

**Fig. 2.**
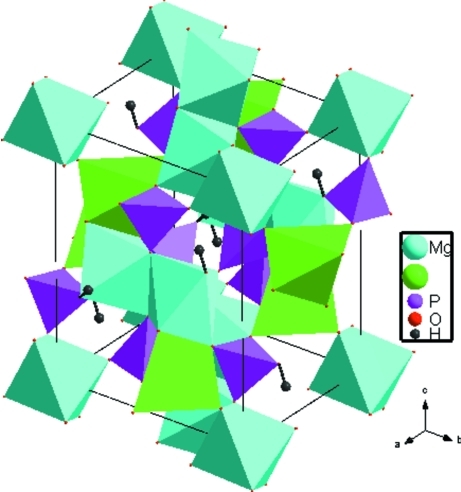
A three-dimensional polyhedral view of the crystal structure of the Mg7(PO4)2(HPO4)4 showing polyhedra linkage.

## supplementary crystallographic information

### Comment

Widespread studies are devoted to the metal phosphate owing to their impressive structural diversity and to their prospective applications in catalysis (Viter & Nagornyi, 2009), ion-exchangers (Clearfield, 1988)) and in batteries performance (Trad *et al.*, (2010)). Mainly, our most attention has been paid to the hydrothermal synthesis of new metal based phosphate. Accordingly, we have recently succeed to obtain new phosphates, such as Ni_2_Sr(PO_4_)_2_ 2H_2_O (Assani *et al.* (2010)), AgMg_3_(PO_4_)(HPO_4_)_2_ (Assani *et al.* (2011*b*)) and Ag_2_Ni_3_(HPO_4_)(PO_4_)_2_ (Assani *et al.* (2011*a*)).

Besides, the investigation of the MO—P_2_O_5_ systems (*M*=divalent cations) has allowed to isolate a new member of the metal phosphates, with a general formula *M*_7_(PO_4_)_2_(HPO_4_)_4_. The present paper aims to develop the hydrothermal synthesis and the structural characterization of the Mg_7_(PO_4_)_2_(HPO_4_)_4_ which is isostructural with Fe_7_(PO_4_)_2_(HPO_4_)_4_ (Zhou *et al.* (2002)), Mn_7_(PO_4_)_2_(HPO_4_)_4_ (Riou *et al.* (1987) and (Rojo *et al.* (2002)), Co_7_(PO_4_)_2_(HPO_4_)_4_ (Lightfoot & Cheetham, (1988)) and with their homologue arsenate Mg_7_(AsO_4_)_2_(HAsO_4_)_4_ (Kolitsch & Bartu, (2004)).

The crystal structure of Mg_7_(PO_4_)_2_(HPO_4_)_4_ is built up from MgO_6_, MgO_4_(OH)_2_ octahedra, MgO_5_ polyhedron, PO_4_ and PO_3_(OH) tetrahedra, sharing corners and edges to form a three-dimensional framework as shown in Fig.1 and Fig.2. In the asymmetric unit, all atoms are in general positions except for atom Mg2, which is located at a crystallographic inversion centre (0, 0, 0). Each OH group is bonded to an Mg and an P atom. Atom Mg2 is located at the centre of an Mg2O_6_ octahedron with significant bond-length distortion as shown in Table 1. In contrast, Mg1O_6_ and Mg3O_4_(OH)_2_ represent less distorted octahedra, and atom Mg4 is surrounded by five O ligands, forming a distorted Mg4O_5_ trigonal bipyramid. In this structure, each Mg1O_6_ and Mg3O_6_ octahedron shares an edge with its symmetrical to form a dimer. Both dimers, Mg1O_10_ and Mg3O_10_ are bound by Mg4O_5_ by sharing two edges to form a zigzag chaine. The Mg2O_6_ octahedron and PO_4_ tetrahedra are linked to neighboring polyhedra by vertices. The three crystallographically independent P atoms show tetrahedral coordination. The PO_4_ groups are relatively regular, although the two protonated groups, centred by P1 and P3, show a stronger angular and bond-length distortion in comparison with the unprotonated P2O_4_ tetrahedron as shown in Table 1. Moreover the OH groups, bridging Mg–(OH)–P, are involved in strong hydrogen bonds (Table 2).

### Experimental

The crystals of the title compound is isolated from the hydrothermal treatment of the reaction mixture of magnesium oxide (MgO) and 85%wt phosphoric acid (H_3_PO_4_) in the nominal proportion corresponding to the molar ratio Mg: P = 7:6. The hydrothermal reaction was conducted in a 23 ml Teflon-lined autoclave, filled to 50% with distilled water and under autogenously pressure at 468 K for two days. After being filtered off, washed with deionized water and air dried, the reaction product consists of a white powder and colourless parallelepipedic crystals corresponding to the title compound.

### Refinement

The  H atoms were initially located in a difference map and refined with O—H distance restraints of 0.86 (1). In a the last cycle they were refined in the riding model approximation with *U*_iso_(H) set to 1.2*U*_eq_(O).

### Crystal data

**Table:**

Mg_7_(PO_4_)_2_(HPO_4_)_4_	*Z* = 1
*M**_r_* = 744.02	*F*(000) = 370
Triclinic, *P*1	*D*_x_ = 2.965 Mg m^−^^3^
Hall symbol: -P 1	Mo *K*α radiation, λ = 0.71073 Å
*a* = 6.4204 (5) Å	Cell parameters from 1923 reflections
*b* = 7.8489 (4) Å	θ = 2.4–27.6°
*c* = 9.4315 (5) Å	µ = 1.06 mm^−^^1^
α = 104.442 (3)°	*T* = 296 K
β = 108.505 (5)°	Parallelepipedic, colourless
γ = 101.189 (8)°	0.16 × 0.10 × 0.07 mm
*V* = 416.70 (4) Å^3^	

### Data collection

**Table:**

Bruker X8 APEX Diffractometer	1923 independent reflections
Radiation source: fine-focus sealed tube	1715 reflections with *I* > 2σ(*I*)
graphite	*R*_int_ = 0.036
φ and ω scans	θ_max_ = 27.6°, θ_min_ = 2.4°
Absorption correction: multi-scan (*SADABS*; Bruker, 2005)	*h* = −8→8
*T*_min_ = 0.881, *T*_max_ = 0.929	*k* = −10→10
9421 measured reflections	*l* = −11→12

### Refinement

**Table:**

Refinement on *F*^2^	Primary atom site location: structure-invariant direct methods
Least-squares matrix: full	Secondary atom site location: difference Fourier map
*R*[*F*^2^ > 2σ(*F*^2^)] = 0.026	Hydrogen site location: difference Fourier map
*wR*(*F*^2^) = 0.072	H-atom parameters constrained
*S* = 1.07	*w* = 1/[σ^2^(*F*_o_^2^) + (0.0325*P*)^2^ + 0.6237*P*] where *P* = (*F*_o_^2^ + 2*F*_c_^2^)/3
1923 reflections	(Δ/σ)_max_ < 0.001
169 parameters	Δρ_max_ = 0.43 e Å^−^^3^
0 restraints	Δρ_min_ = −0.42 e Å^−^^3^

### Special details

**Table:**

Geometry. All s.u.'s (except the s.u. in the dihedral angle between two l.s. planes) are estimated using the full covariance matrix. The cell s.u.'s are taken into account individually in the estimation of s.u.'s in distances, angles and torsion angles; correlations between s.u.'s in cell parameters are only used when they are defined by crystal symmetry. An approximate (isotropic) treatment of cell s.u.'s is used for estimating s.u.'s involving l.s. planes.
Refinement. Refinement of *F*^2^ against all reflections. The weighted *R*-factor *wR* and goodness of fit *S* are based on *F*^2^, conventional *R*-factors *R* are based on *F*, with *F* set to zero for negative *F*^2^. The threshold expression of *F*^2^ > σ(*F*^2^) is used only for calculating *R*-factors(gt) *etc*. and is not relevant to the choice of reflections for refinement. *R*-factors based on *F*^2^ are statistically about twice as large as those based on *F*, and *R*- factors based on all data will be even larger.

### Fractional atomic coordinates and isotropic or equivalent isotropic displacement parameters (Å^2^)

**Table:**

	*x*	*y*	*z*	*U*_iso_*/*U*_eq_	
Mg1	−0.38348 (12)	0.54390 (10)	−0.10950 (8)	0.00639 (17)	
Mg2	0.0000	0.0000	0.0000	0.0094 (2)	
Mg3	−0.05324 (13)	0.28758 (10)	−0.51536 (9)	0.00821 (17)	
Mg4	−0.27778 (13)	0.19081 (11)	−0.28530 (9)	0.00902 (17)	
P1	0.22691 (9)	0.14512 (8)	−0.22413 (6)	0.00561 (13)	
P2	0.08899 (9)	0.58025 (7)	−0.17255 (6)	0.00465 (13)	
P3	−0.59090 (9)	0.23141 (8)	−0.62865 (7)	0.00658 (14)	
O1	0.0189 (3)	0.1762 (2)	−0.33779 (18)	0.0081 (3)	
O2	0.2208 (3)	0.1877 (2)	−0.05694 (18)	0.0074 (3)	
O3	0.4517 (3)	0.2446 (2)	−0.22968 (18)	0.0077 (3)	
O4	0.2013 (3)	−0.0667 (2)	−0.27972 (18)	0.0091 (3)	
H4	0.1828	−0.1148	−0.2104	0.011*	
O5	0.3069 (3)	0.5385 (2)	−0.08555 (18)	0.0068 (3)	
O6	0.0589 (3)	0.5452 (2)	−0.34643 (18)	0.0073 (3)	
O7	0.1098 (3)	0.7857 (2)	−0.09645 (19)	0.0094 (3)	
O8	−0.1240 (3)	0.4602 (2)	−0.16487 (18)	0.0070 (3)	
O9	−0.3803 (3)	0.2112 (2)	−0.50853 (19)	0.0104 (3)	
O10	−0.5267 (3)	0.3827 (2)	−0.69363 (19)	0.0106 (3)	
O11	−0.7360 (3)	0.0488 (2)	−0.76029 (19)	0.0097 (3)	
O12	−0.7344 (3)	0.2950 (2)	−0.52719 (19)	0.0111 (3)	
H12	−0.6495	0.3935	−0.4485	0.013*	

### Atomic displacement parameters (Å^2^)

**Table:**

	*U*^11^	*U*^22^	*U*^33^	*U*^12^	*U*^13^	*U*^23^
Mg1	0.0064 (4)	0.0077 (4)	0.0058 (4)	0.0022 (3)	0.0028 (3)	0.0028 (3)
Mg2	0.0120 (5)	0.0086 (5)	0.0095 (5)	0.0035 (4)	0.0061 (4)	0.0034 (4)
Mg3	0.0091 (4)	0.0083 (4)	0.0076 (4)	0.0021 (3)	0.0037 (3)	0.0029 (3)
Mg4	0.0093 (4)	0.0082 (4)	0.0104 (4)	0.0027 (3)	0.0047 (3)	0.0031 (3)
P1	0.0055 (3)	0.0055 (3)	0.0056 (3)	0.0011 (2)	0.0024 (2)	0.0016 (2)
P2	0.0045 (3)	0.0052 (3)	0.0047 (3)	0.0017 (2)	0.0019 (2)	0.0020 (2)
P3	0.0058 (3)	0.0073 (3)	0.0067 (3)	0.0019 (2)	0.0023 (2)	0.0025 (2)
O1	0.0056 (7)	0.0103 (8)	0.0084 (8)	0.0024 (6)	0.0019 (6)	0.0041 (6)
O2	0.0086 (7)	0.0070 (7)	0.0056 (7)	0.0010 (6)	0.0031 (6)	0.0008 (6)
O3	0.0056 (7)	0.0078 (7)	0.0088 (7)	−0.0002 (6)	0.0037 (6)	0.0019 (6)
O4	0.0133 (8)	0.0067 (7)	0.0085 (8)	0.0025 (6)	0.0057 (6)	0.0030 (6)
O5	0.0053 (7)	0.0094 (7)	0.0068 (7)	0.0034 (6)	0.0024 (6)	0.0034 (6)
O6	0.0097 (7)	0.0081 (7)	0.0053 (7)	0.0036 (6)	0.0035 (6)	0.0030 (6)
O7	0.0119 (8)	0.0060 (7)	0.0109 (8)	0.0038 (6)	0.0050 (6)	0.0021 (6)
O8	0.0049 (7)	0.0071 (7)	0.0091 (7)	0.0010 (6)	0.0037 (6)	0.0020 (6)
O9	0.0085 (8)	0.0157 (8)	0.0086 (8)	0.0059 (6)	0.0030 (6)	0.0052 (6)
O10	0.0117 (8)	0.0093 (8)	0.0087 (8)	−0.0002 (6)	0.0018 (6)	0.0048 (6)
O11	0.0098 (7)	0.0073 (7)	0.0102 (8)	0.0014 (6)	0.0025 (6)	0.0025 (6)
O12	0.0084 (8)	0.0137 (8)	0.0099 (8)	0.0037 (6)	0.0042 (6)	0.0010 (6)

### Geometric parameters (Å, °)

**Table:**

Mg1—O10^i^	2.0235 (17)	P1—O2	1.5431 (16)
Mg1—O5^ii^	2.0462 (17)	P1—O4	1.5718 (16)
Mg1—O5^iii^	2.0643 (17)	P2—O5	1.5237 (16)
Mg1—O8	2.0698 (17)	P2—O6	1.5350 (16)
Mg1—O2^ii^	2.1093 (17)	P2—O8	1.5362 (16)
Mg1—O3^iii^	2.2065 (17)	P2—O7	1.5533 (16)
Mg2—O7^iv^	2.0630 (16)	P3—O10	1.5131 (16)
Mg2—O7^ii^	2.0630 (16)	P3—O11	1.5245 (17)
Mg2—O2	2.1296 (15)	P3—O9	1.5287 (16)
Mg2—O2^v^	2.1296 (15)	P3—O12	1.5853 (17)
Mg2—O11^vi^	2.2395 (16)	O2—Mg1^ii^	2.1093 (17)
Mg2—O11^vii^	2.2395 (16)	O3—Mg4^x^	2.0549 (17)
Mg3—O4^viii^	2.0415 (17)	O3—Mg1^x^	2.2065 (17)
Mg3—O1	2.0443 (17)	O4—Mg3^viii^	2.0415 (17)
Mg3—O6	2.0606 (17)	O4—H4	0.8601
Mg3—O6^ix^	2.0649 (17)	O5—Mg1^ii^	2.0462 (17)
Mg3—O12^x^	2.0759 (17)	O5—Mg1^x^	2.0643 (17)
Mg3—O9	2.0954 (17)	O6—Mg3^ix^	2.0649 (17)
Mg4—O8	2.0041 (17)	O7—Mg2^xi^	2.0630 (16)
Mg4—O11^vi^	2.0426 (18)	O10—Mg1^i^	2.0235 (17)
Mg4—O9	2.0544 (17)	O11—Mg4^vi^	2.0426 (18)
Mg4—O3^iii^	2.0549 (17)	O11—Mg2^xii^	2.2395 (16)
Mg4—O1	2.1312 (17)	O12—Mg3^iii^	2.0759 (17)
P1—O3	1.5252 (16)	O12—H12	0.8600
P1—O1	1.5297 (16)		
			
O10^i^—Mg1—O5^ii^	177.45 (7)	O1—Mg3—O12^x^	90.91 (7)
O10^i^—Mg1—O5^iii^	93.60 (7)	O6—Mg3—O12^x^	95.01 (7)
O5^ii^—Mg1—O5^iii^	84.54 (7)	O6^ix^—Mg3—O12^x^	82.93 (7)
O10^i^—Mg1—O8	89.62 (7)	O4^viii^—Mg3—O9	82.41 (7)
O5^ii^—Mg1—O8	91.62 (7)	O1—Mg3—O9	80.21 (7)
O5^iii^—Mg1—O8	161.77 (7)	O6—Mg3—O9	96.14 (7)
O10^i^—Mg1—O2^ii^	97.16 (7)	O6^ix^—Mg3—O9	108.15 (7)
O5^ii^—Mg1—O2^ii^	84.67 (7)	O12^x^—Mg3—O9	165.63 (8)
O5^iii^—Mg1—O2^ii^	92.32 (7)	O8—Mg4—O11^vi^	135.86 (7)
O8—Mg1—O2^ii^	105.09 (7)	O8—Mg4—O9	97.00 (7)
O10^i^—Mg1—O3^iii^	96.93 (7)	O11^vi^—Mg4—O9	124.27 (7)
O5^ii^—Mg1—O3^iii^	81.15 (6)	O8—Mg4—O3^iii^	83.43 (7)
O5^iii^—Mg1—O3^iii^	83.53 (6)	O11^vi^—Mg4—O3^iii^	102.94 (7)
O8—Mg1—O3^iii^	78.27 (6)	O9—Mg4—O3^iii^	98.66 (7)
O2^ii^—Mg1—O3^iii^	165.53 (7)	O8—Mg4—O1	88.96 (7)
O7^iv^—Mg2—O7^ii^	180.00 (7)	O11^vi^—Mg4—O1	84.69 (7)
O7^iv^—Mg2—O2	91.18 (6)	O9—Mg4—O1	79.14 (7)
O7^ii^—Mg2—O2	88.82 (6)	O3^iii^—Mg4—O1	171.78 (7)
O7^iv^—Mg2—O2^v^	88.82 (6)	O3—P1—O1	111.79 (9)
O7^ii^—Mg2—O2^v^	91.18 (6)	O3—P1—O2	114.92 (9)
O2—Mg2—O2^v^	180.00 (7)	O1—P1—O2	110.31 (9)
O7^iv^—Mg2—O11^vi^	90.17 (6)	O3—P1—O4	106.96 (9)
O7^ii^—Mg2—O11^vi^	89.83 (6)	O1—P1—O4	107.87 (9)
O2—Mg2—O11^vi^	85.85 (6)	O2—P1—O4	104.43 (9)
O2^v^—Mg2—O11^vi^	94.15 (6)	O5—P2—O6	109.03 (9)
O7^iv^—Mg2—O11^vii^	89.83 (6)	O5—P2—O8	111.40 (9)
O7^ii^—Mg2—O11^vii^	90.17 (6)	O6—P2—O8	109.62 (9)
O2—Mg2—O11^vii^	94.15 (6)	O5—P2—O7	109.95 (9)
O2^v^—Mg2—O11^vii^	85.85 (6)	O6—P2—O7	108.49 (9)
O11^vi^—Mg2—O11^vii^	180.00 (8)	O8—P2—O7	108.30 (9)
O4^viii^—Mg3—O1	104.92 (7)	O10—P3—O11	112.06 (9)
O4^viii^—Mg3—O6	165.27 (8)	O10—P3—O9	112.08 (9)
O1—Mg3—O6	89.19 (7)	O11—P3—O9	111.85 (9)
O4^viii^—Mg3—O6^ix^	87.80 (7)	O10—P3—O12	107.28 (9)
O1—Mg3—O6^ix^	165.83 (8)	O11—P3—O12	109.21 (9)
O6—Mg3—O6^ix^	78.70 (7)	O9—P3—O12	103.90 (9)
O4^viii^—Mg3—O12^x^	89.06 (7)		

Symmetry codes: (i) −*x*−1, −*y*+1, −*z*−1; (ii) −*x*, −*y*+1, −*z*; (iii) *x*−1, *y*, *z*; (iv) *x*, *y*−1, *z*; (v) −*x*, −*y*, −*z*; (vi) −*x*−1, −*y*, −*z*−1; (vii) *x*+1, *y*, *z*+1; (viii) −*x*, −*y*, −*z*−1; (ix) −*x*, −*y*+1, −*z*−1; (x) *x*+1, *y*, *z*; (xi) *x*, *y*+1, *z*; (xii) *x*−1, *y*, *z*−1.

### Hydrogen-bond geometry (Å, °)

**Table:**

*D*—H···*A*	*D*—H	H···*A*	*D*···*A*	*D*—H···*A*
O4—H4···O7^iv^	0.86	1.61	2.460 (2)	172.
O12—H12···O10^i^	0.86	1.80	2.656 (2)	171.

Symmetry codes: (iv) *x*, *y*−1, *z*; (i) −*x*−1, −*y*+1, −*z*−1.
